# Impact of brand equity and service quality on the reputation of universities and students’ intention to choose them: The case of IIUM and UIN

**DOI:** 10.12688/f1000research.122386.1

**Published:** 2022-11-30

**Authors:** Sylvia Rozza Rizard, Bambang Waluyo, Irwandi Jaswir

**Affiliations:** 1 Accounting, Politeknik Negeri Jakarta, Depok, West Java, Indonesia; 2 International Islamic University Malaysia, Selangor, Malaysia

**Keywords:** brand equity, price, service quality, students’ intention, university’s reputation

## Abstract

**Background: **Numerous studies have been carried out on the impacts of brand equity and service quality of higher education institutions (HEIs) on their reputation and students’ satisfaction. This research aimed to compare the impact of brand equity and service quality on universities’ reputations, namely Universitas Islam Negeri (UIN) in Indonesia and International Islamic University Malaysia (IIUM) in Malaysia, and Indonesian students’ intention to choose the universities, which is moderated by study expense (price). UIN and IIUM are HEIs with a similar university concept, and Indonesian students have recently shown a high interest in them. The two universities have faculties not only in the field of Islamic studies but in general fields of studies as well, which are usually held by non-Islamic Universities. Therefore, their competitiveness against non-Islamic universities, especially the University of Indonesia (UI) has increased.

**Methods:** The statistical measurement tool used was structural equation modeling (SEM). The number of items stated in the questionnaire was 45. Therefore, minimum data to be collected were 5 × 45 or 225 which rounded up to 228 from Indonesian students at UIN and IIUM (114 UIN students, and 114 Indonesian student respondents from IIUM).

**Results: **The study results show that the universities’ reputations are strongly affected by their brand equity and service quality, which then affect students’ intention to choose the universities. Students had a higher intention to choose IIUM than UIN. The limitation of this research is that the effect of study expense on the intention of Indonesian students to study at UIN or IIUM has not yet been conducted. It will be conducted in the next study.

**Conclusions: **These results are expected to be useful to UIN, IIUM, and especially Politeknik Negeri Jakarta (PNJ) in determining a strategy to enhance their reputations and the intention of Indonesian students to study there.

## Introduction

One form of higher education institution (HEI) in Indonesia is Institut Agama Islam Negeri (IAIN), which is an Islamic university that provides academic education in Islamic religious disciplines. The first IAIN was established in Yogyakarta in 1960. In 2002, IAIN Syarif Hidayatullah in Jakarta was changed to a State Islamic University, namely Universitas Islam Negeri (UIN). Many other IAINs were established throughout Indonesia, until 2019. The UIN format allows universities to open faculties or departments in other fields of study outside of Islamic studies. The impact of these changes is the increase in the number of universities that offer majors in faculties of non-Islamic studies that can be chosen by prospective students. Several faculties or departments that usually only exist in non-Islamic study universities, such as economics, psychology, biology, pharmacy, medicine, electrical engineering, mechanical engineering, and civil engineering are currently also fields of studies offered at UIN. Consequently, UIN has become a competitor of non-Islamic study universities as an alternative choice of institution for prospective students to attend. The number of students who are interested in studying at UIN continues to increase, while the number of students who enrolled at the University of Indonesia (UI), the biggest competitor of UIN, has decreased ( https://pddikti.kemdikbud.go.id/perbandingan/perguruan).

From the numerous studies done in this context, it can be concluded that students’ satisfaction or loyalty to a university is influenced by factors such as brand equity, and the service quality of the university. Both of these variables have an impact on a university’s reputation ( Ali M., and Ahmed M., 2018; Amal S. A, Shurair, and Pokharel S., 2019; Arian M. and Khoshtaria, T 2020). So, reputation is an important factor that determines satisfaction or loyalty ( Aidah, Supardi, Juhji; 2020; Pinar, M., Girard, T., and Basfirinci C., 2020; Polkinghorne, M., Roushan, G., and Taylor, J. 2017). However, this research was conducted with different research models, to measure the effect of brand equity and service quality on universities’ reputations and prospective students’ intention to choose them. The researchers could not find this type of research framework.

Another factor considered is the study expense (tuition fee + living cost) at the selected universities ( McGettigan, A. 2013; Palfreymam, D., and Tapper, T., 2016). Since the birth of UIN, there has been a decrease in the number of prospective students choose non-Islamic universities. This is because UIN also offers academic courses that were previously only offered at non-Islamic universities. The reputation of UIN is quite good, and its study expense is lower. This is shown by the accreditation of many of UIN’s study programs bestowed by The National Accreditation Board (BAN-PT) as grade “A”. So, the number of interested students who study at UIN has increased.

One of the universities with an Islamic concept in neighboring country Malaysia is the International Islamic University Malaysia (IIUM). This university is also a competitor of non-Islamic universities and UIN in Indonesia. The number of Indonesian students who are interested in studying at IIUM is also increasing. In choosing a place to study in Malaysia, prospective Indonesian students also consider the same factors that are applicable in Indonesia, which are moderated by study expenses in that country ( Zeithaml, A., 1988).

This study aimed to compare the intention of Indonesian students to choose UIN or IIUM as a place to study. The researchers have not been able to find research in Indonesia that examines the impact of a university’s reputation based on its brand equity and service quality on students’ intention to choose it. The results of this study are expected to be useful to UIN, and IIUM in formulating policies to determine factors that affect their reputation and students' intentions to choose them. The results will also be useful to Indonesian universities in making the same policies and strategies. This study is also applicable to Jakarta State Polytechnic (PNJ), who funded this research in formulating strategies to help PNJ gain a better reputation. It will result in a higher number of quality graduates, and ultimately increases the competitiveness of graduates, which is one of the visions of PNJ.

### Literature review

Students are customers ( Royo, 2017), in the marketing concept. To study in a university, sometimes they pay a lot of money; expensive tuition fees are usually correlated with the quality of education at the university. For this reason, they deserve all the best services from the university ( Mehrtens, I., 2016). Nuriah and Rahma (2018) examined the influence of a university’s reputation on students' decisions to choose the university. They concluded that the reputation of a university reflects the quality of the university so that it becomes a determinant of students' choices for it. Mourad, Ennew, Kortam (2011), examined the effect of word of mouth (WOM) and a university’s reputation on students' decisions to choose a university. They concluded that WOM had no effect on students’ decisions in choosing a university, but the university’s reputation did. Mwiya, Bwalya, Siachinji, Sikombe, Chanda, and Chawala (2017), conducted research in Zambia on the effect of the quality of higher education services on students’ satisfaction and behavioral intentions in the form of positive loyalty and WOM. The results of this study indicate that all dimensions of the quality of educational services have a significant positive effect on students’ satisfaction, and ultimately affect their loyalty and willingness to spread positive WOM.

Besides the above objectives, this research was also conducted to support the implementation of PNJ's Strategic Planning of Research (RENSTRA), where the research roadmap for the 2019-2025 period focuses on the market. The market is defined as the collection of actual and potential buyers of a product. While the product is anything that can be offered to the market that can satisfy wants and needs ( Kotler, P., and Armstrong, G., 2014). The market in the context of this research is customers or students studying at the university. This study measured the influence of brand equity and quality of educational services on the reputation of a university, as well as students' intentions to choose a university. Brand equity, service quality, and companies’ reputation are intangible market-based assets that affect customers’ satisfaction ( Almarri, Khalid and Gardiner, Paul, 2014; Walsh and Beatty, R. Sharon, 2007). In the context of PNJ as a HEI, this also applies.

From relevant studies that have been carried out, no research has compared the effect of brand equity and service quality on the reputation of the two universities (from Indonesia and Malaysia) with similar concepts and the intentions of Indonesian students to study at either university, which are currently in high demand.

In this study, four constructs wereproposed, namely brand equity, service quality, companies’ reputation, and price. Their impact on customers’ intentions was analyzed. Brand equity and service quality directly affect a company's reputation. Furthermore, the reputation of a company that is moderated by price will have an impact on customers’ intentions. These constructs are explained as follows.

### Brand equity

Brand equity is one of the most prized assets of firms and a key concept for marketing academics ( Ambler, 2003; Christodoulides and de Chernatony, 2010; Christodoulides, Cadogan, J. and Veloutsou, V., 2015). Although brand equity has been extensively researched in the context of physical products, less attention has been devoted to understanding the concept in relation to a service sector context ( Mourad, *et al.*, 2011). Świtała, Gamrot, Reformat, and Reformat (2018) said that numerous definitions explaining the essence of the brand have been provided in the literature over the last twenty years ( Kall, 2005; Kapferer, 2008; Keller, 2011; Witek-Hejduk, 2011). Brand equity is defined as a set of assets connected to the brand name and add value to the product/service for customers ( Dennis, S., Alamanos, E. and Bourlakis, M. 2016). Arian M. and Tornike Khoshtaria, (2020) said that brand equity affects the reputation of a company. Keller, K. (2013), Schiffman, LG. and Kanuk, L. (2004), defined brand equity as a different response from consumers to a good brand compared to unbranded products when both have the same marketing stimuli and attributes. Meanwhile, Priporas, V., and Kamenidou, I. (2011), and Yoo, and Donthu (2001) defined brand equity from the customer's perspective as the differential effect of brand knowledge on consumers’ response to the marketing of the brand. From these various definitions, the consensual definition is that brand equity increases the value of a product because of its brand name ( Khanna, M., Jacob, I., and Yadav, N. 2015). The concept of brand equity is of particular relevance to consumers’ choices ( Mourad, *et al.*, 2011). The brand equity dimension in this matter is brand awareness which consists of brand recognition, brand recall, and brand image which means strong, favorable, and unique associations of the brand in the customer’s memory ( Keller, 2013). Pinar, M., Trapp, P., Girard, T., and Boyt, T. (2013), Pinar, M., Trapp, P., Girard, T., and Boyt, T. (2011), Walsh and Beatty, R. Sharon, (2007) explained that there are four brand dimensions, namely brand loyalty, brand awareness, perceived quality, and brand associations ( Sapna and Sheetal 2016). This study aimed to measure the impact of brand equity on the reputation of the university ( Lomer, S., Papatsiba, V., and Naidoo, R. 2018; Chapelo, C., 2015).

### Service quality

Service quality is the totality of the characteristics of goods and services that show their ability to satisfy customers’ needs, both obvious and hidden. For companies engaged in the service sector, providing quality services to customers is an absolute requirement if they want to achieve success ( Kotler, P., and Armstrong, G., 2014). Another definition of service quality is the effort to fulfill the needs and desires of consumers and the accuracy of their delivery in balancing consumers’ expectations. Dimensions of service quality are reliability, responsiveness, assurance, empathy, and tangibility ( Zeithaml, A. V., Bitner J. M., and Gremler D. D., 2013). In this study, the quality of educational services affected the reputation of the university ( Gronroos, C. Kamalanabhan, T.J., and Seebaluck, A.K., 2019; Polkinghorne **,** M., Roushan, G., and Taylor, J. 2017).

### Corporate reputation

A company’s reputation is defined as a collective representation that shows the company's position internally to its employees, and externally to its stakeholders ( Arian M. and Tornike Khoshtaria, 2020). Another definition of corporate reputation is knowledge of a company's actions and the results of the crystallization of the company's ability to deliver valuable outcomes to its stakeholders ( Nuriah and Rahma, 2018). Corporate reputation is customers' overall evaluation of a company based on their reactions to products, services, communication activities, and interaction with the company’s representations and/or known activities ( Walsh and Beatty, 2007). Corporate reputation dimensions in this context include customers’ orientation, being a good employer, being a reliable and financially strong company, product and service quality, and social and environmental responsibility ( Walsh and Beatty, 2007). A company’s reputation is a critical construct interpreted as the stakeholder’s perception of the company as a whole ( Walsh and Wiedmann, 2004). The reputation of the company in this study was the reputation of the university chosen by students as an antecedent of their choice.

### Price

Price is the amount of value that customers exchange for having or benefiting from having or using a product or service ( Kotler and Armstrong, 2014; Zeithaml, A., 1988; Palfreymam, D., and Tapper, T., 2016). Dimensions of price are affordability, price competition, compatibility of price and quality of product, and compatibility of price and benefit of product ( Kotler and and Armstrong, 2014). In the context of studying at a university, price means study expense which consists of tuition fees and living costs incurred. Price will moderate the influence of a company’s reputation and students' intention to choose a university ( McGettigan, A., 2013).

### Customers’ intention

Intention or interest is a high inclination of one’s heart towards something; excitement, desire. Buying interest is the stage of a customer's tendency to act before the buying decision is actually implemented ( Chong, Y.S. and Ahmed, P.K. 2015; Constantinides and Stagno, 2012). According to Ali M., and Ahmed M. (2018) and Amal S. A, Shurair and Shaligram Pokharel (2019), the intention is a consumer's interest in a product by seeking additional information. Consumers’ buying interest entails a consumer having a desire to buy or choose a product, based on experience in choosing, using and consuming, or even wanting a product. Satisfaction in buying goods or services strengthens interest in buying. Dissatisfaction usually eliminates interest. Customers’ satisfaction has been seen to produce four outcomes for shareholders, in this case, the university concerned ( Arian M. and Tornike Khoshtaria, 2020): 1. Satisfied customers will be encouraged to buy more from the company. 2. Satisfied customers will buy various products from the company. In the context of a university, satisfied students will be interested in continuing their studies at the same university. 3. Recommendations and positive WOM can also be carried out by satisfied customers, which will increase the value of the university. Dimensions of customers’ intention are transactional intention, referral intention, preferential intention, and explorative intention ( Schiffman and Kanuk, 2004). Satisfied students will recommend the university where they studied to their relatives or other people. 4. Customers who are increasingly satisfied will provide opportunities for a company to increase prices, thereby increasing the value of the company. Satisfied students will provide opportunities for universities to increase their tuition fees, making it more profitable for the universities. Harahap, Hurriyati, Gaffar and Amanah (2018) said that the reputation of a university strongly affects the intention to choose the university.

## Methods

### Ethical considerations

The study samples were Indonesian students attending two well-known universities namely Universitas Islam Negeri (UIN) Syarif Hidayatullah in Jakarta, an education provider using both Islamic and non-Islamic concepts, and Indonesian students studying at International Islamic University Malaysia (IIUM) in Malaysia. The population of the research was Indonesian students at the two universities. Due to the lack of an Institutional Review Board at the researchers’ institution, no formal ethical approval could be obtained for this research. However, this research is considered low-risk due to the scope of the survey and nature of data collected, i.e. factors affecting Indonesian students’ choice of university in Indonesia. The Director of Politeknik Negeri Jakarta (PNJ) approved the research beforehand and informed the respondents that responses would be kept anonymous and published only to support this study, and written informed consent was obtained from participants.

### Sample population

**Figure 1.**
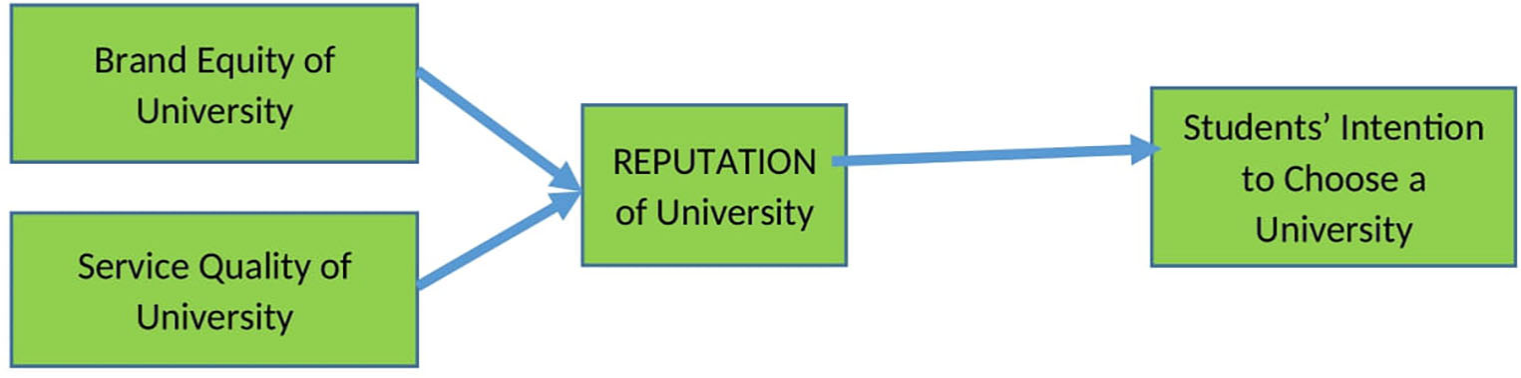
Research framework.

This research was planned to last for two years. Measuring the effect of brand equity and service quality of the universities on their reputation and the intention of Indonesian students to choose the universities was carried out in the first year. Furthermore, the moderation of study expenses on the influence of the universities’ reputation and students' intention to choose the universities was measured in the second year. The framework of this research is shown below ( Figure 1).

The study subjects were Indonesian students studying at two well-known universities, namely UIN Syarif Hidayatullah in Jakarta, and Indonesian students studying at IIUM. The population of the research was Indonesian students at the two universities. Non-probability sampling method was used to select the respondents as indicated in the questionnaire. The statistical measurement tool used is Structural Equation Modeling (SEM) ( Wijanto, 2008), stated that number of respondents should be collected is at least 5 × number of items in the questionnaire. The number of statement items stated in the questionnaire was 45. Therefore, data were collected from 5 × 45 or 225 which rounded up to 228 Indonesian students at UIN and IIUM. 2 PNJ students worked as research assistants collected the data from 114 UIN students, and the other 2 did it from 114 Indonesian student respondents from IIUM. The questionnaire was compiled from the dimensions of each construct and then derived into various indicators.

## Results

A two-step approach was used in analyzing the research model ( Anderson and Gerbing, 1988). In the first step, the analysis was done on the measurement model. This was done to check whether the measurement model has good validity and reliability in the sample data used. The second step entails adding a structural path based on the hypotheses of the measurement model to produce the hybrid model. The model and structural relations proposed in the hypotheses were analyzed and evaluated. The measurement model has 4 latent variables namely brand equity, service quality, higher education reputation, and customers’/students’ intention to study at the universities.

The validity of the measurement model was seen from the standardized loading factor from each indicator of variables by checking its significance with its latent variables. The standardized loading factor used was ≥ 0.5 ( Igbaria, Zinatelli, Cragg and Cavaye, 1997). Reliability was measured in average variance extracted (AVE), and composite reliability (CR). The reliability of a good measurement model is CR ≥ 0.70 and AVE ≥ 0.50. CR and AVE were taken from standardized loading factors and error variances ( Wijanto, 2008).

**Figure 2.**
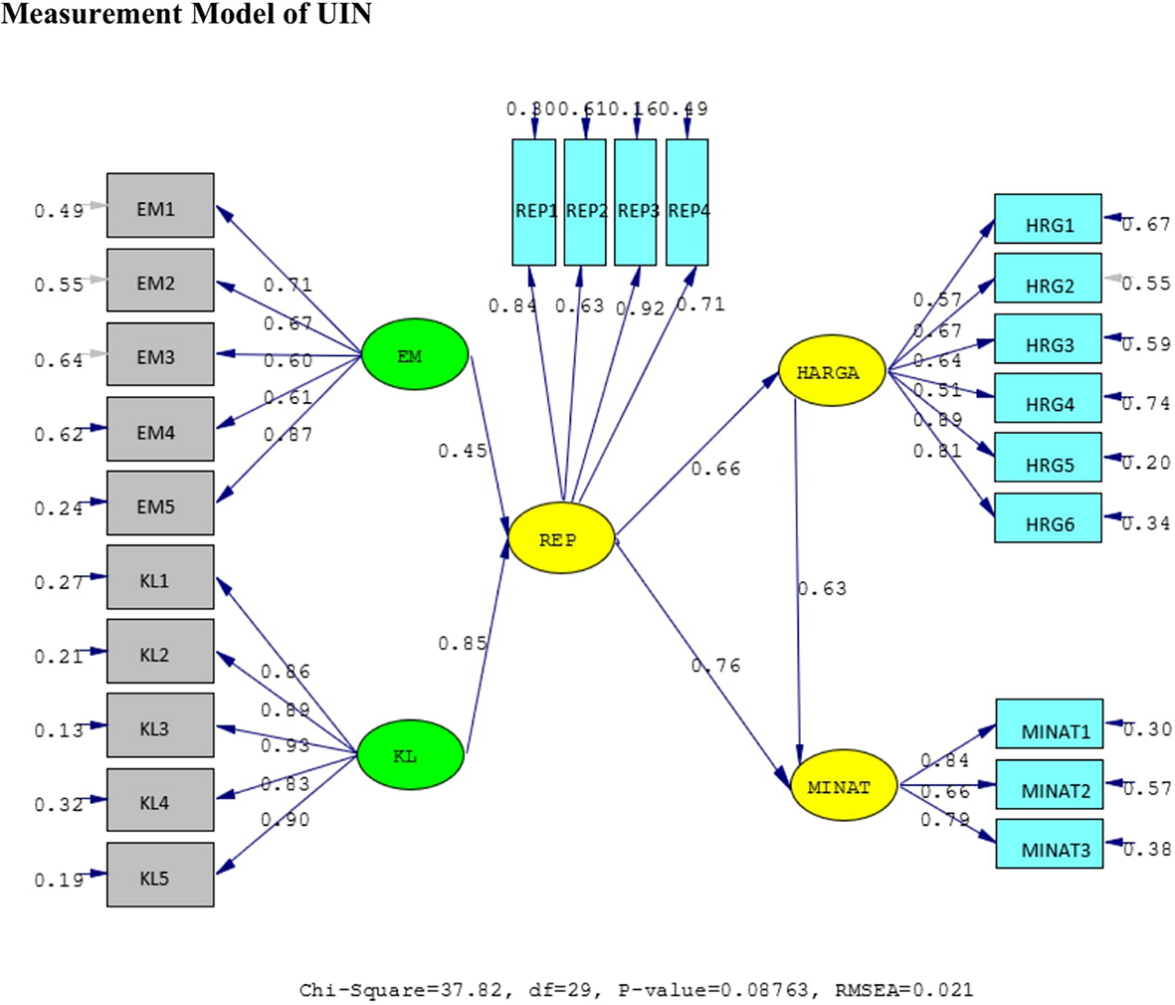
Measurement Model of UIN.

**Figure 3.**
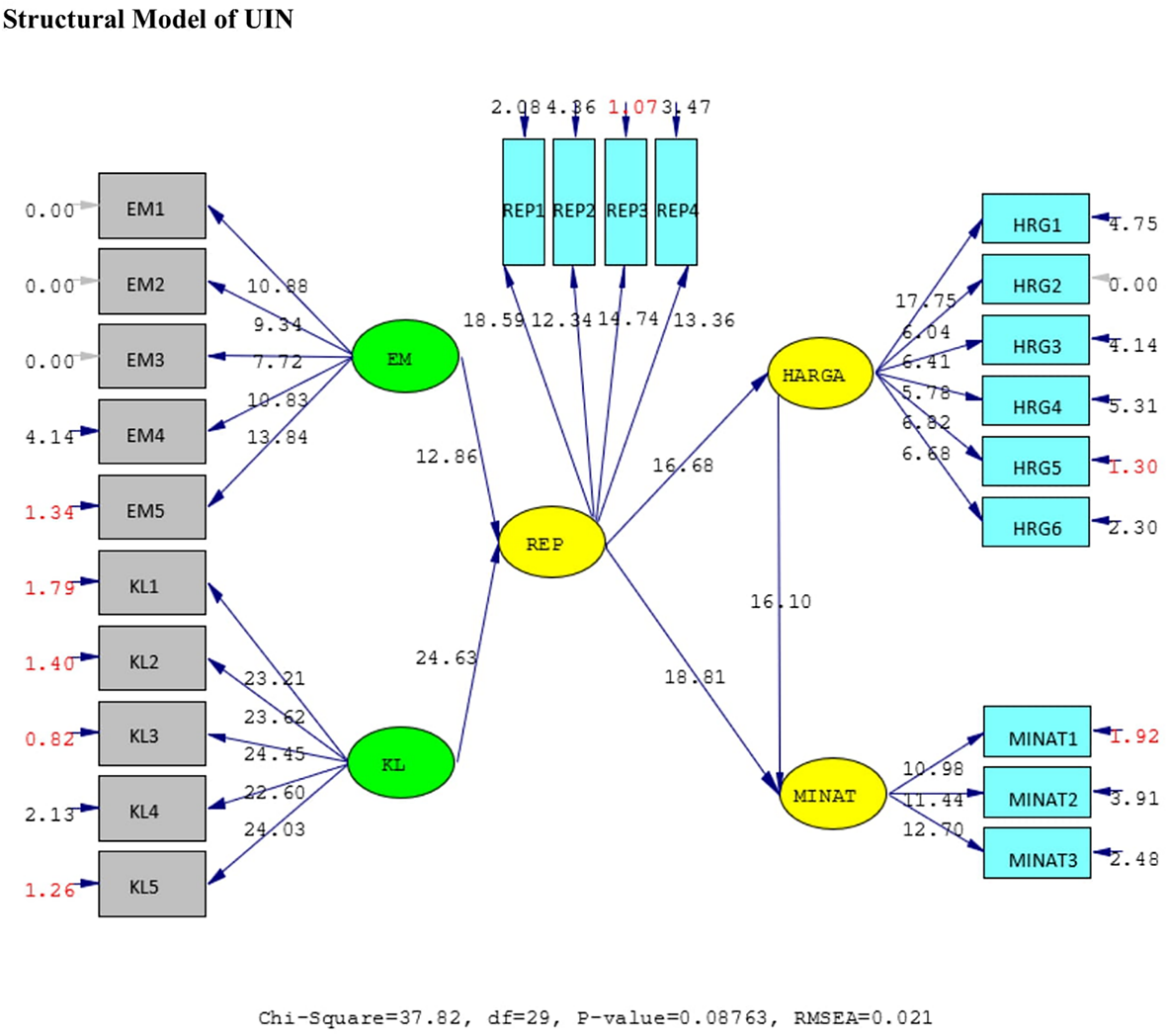
Structural model of Universitas Islam Negeri (UIN). Remark: EM = BE (Brand Equity), KL = SQ (Service Quality), REP = Reputation, HRG = P (Price), Minat = I (Intention).

**Figure 4.**
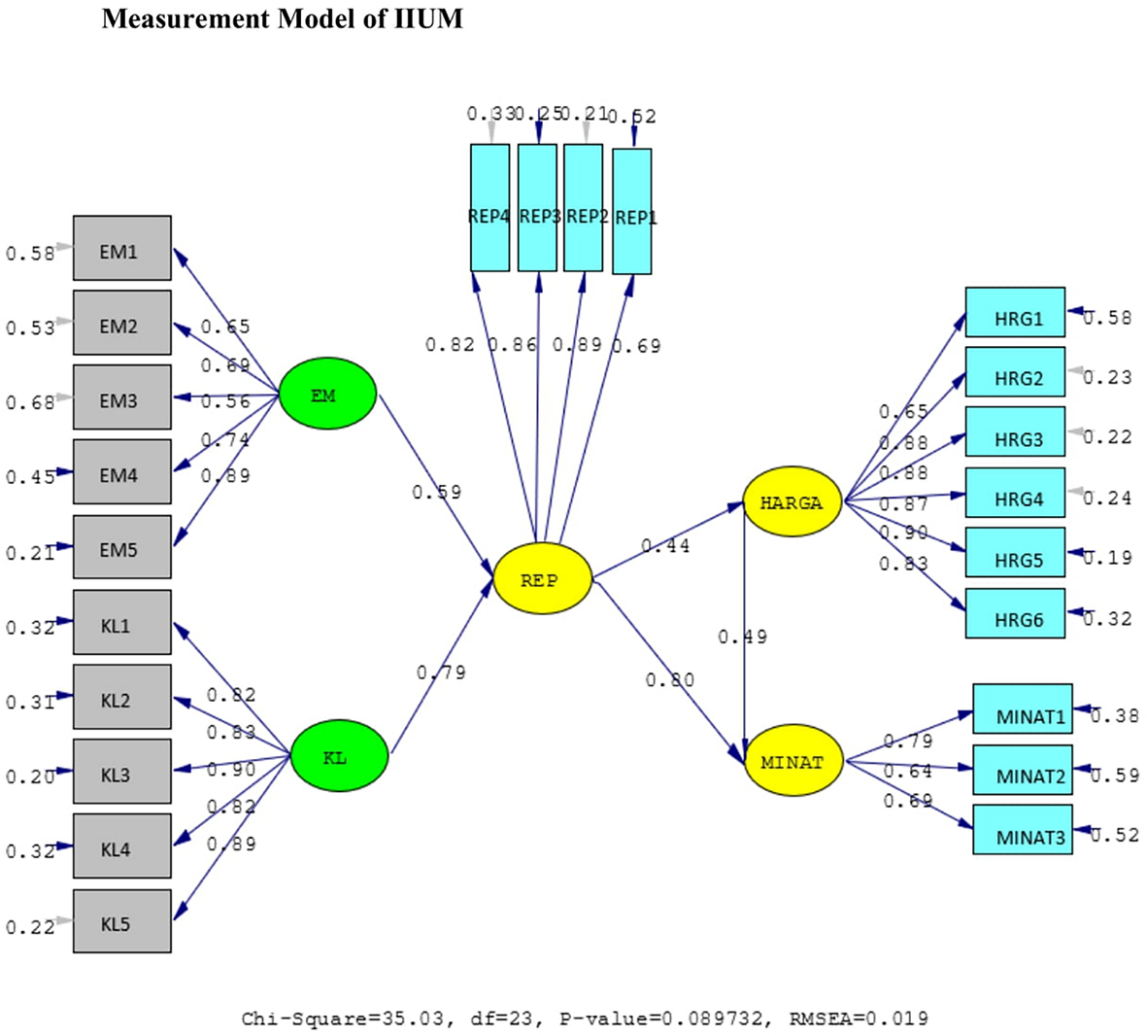
Measurement model of Internationa Islamic University Malaysia (IIUM). Remark: EM = BE (Brand Equity), KL = SQ (Service Quality), REP = Reputation, HRG = P (Price), Minat = I (Intention).

**Figure 5.**
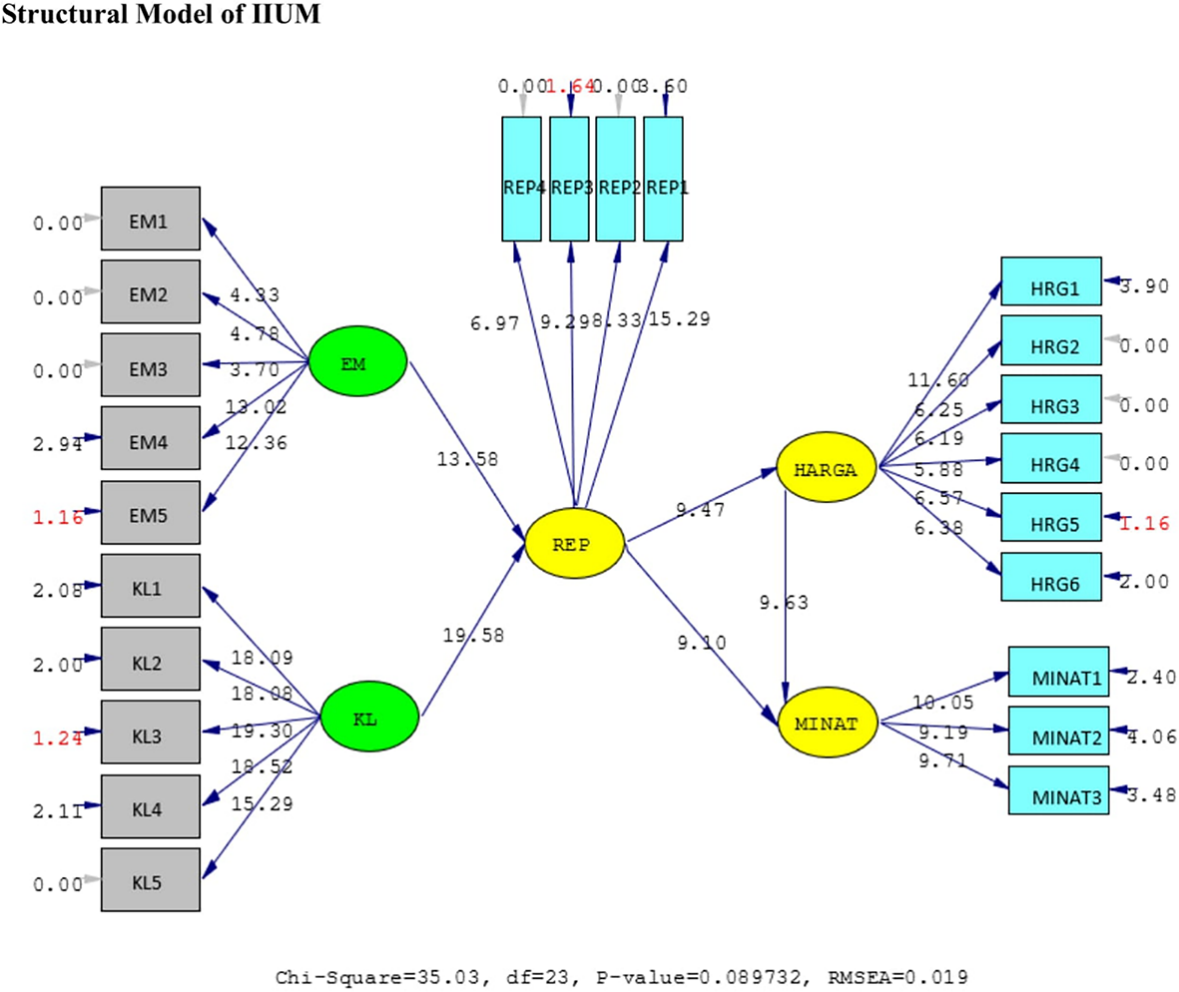
Structural model of International Islamic University Malaysia (IIUM). Remark: EM = Ekuitas Merek (Brand Equity = BE), KL = Kualitas Layanan (Service Quality = SQ), REP = Reputation, HRG = Harga (Price = P), Minat = Intention = I.

The results of the validity test show that the answers of the UIN and IIUM respondents to all the statement items have a validity coefficient greater than r-critical 0.3. The answers to these items are feasible or valid. Reliability testing is carried out on the statement items that are included in the valid category. This result indicates that the statement items are reliable, as shown by standardized loading factors in Table 1 below. All the indicators of the variables have a *standardized loading factor* value > 0.5. All measured variables have good validity and reliability value with AVE > 0.5 and CR > 0.7. The Measurement model and Structural model of UIN can be seen in Figures 2 and 3, while, the Measurement model and Structural model of IIUM can be seen in Figures 4 and 5.

**Table 1.** Validity and reliability of data from Universitas Islam Negeri (UIN) and International Islamic University Malaysia (IIUM).

Indicator	UIN					IIUM				
	SLF	ei	T-stats	CR	VE	SLF	ei	T-stats	CR	VE
EM1	0.71	0.49	10.88	0.8250	0.5902	0.65	0.58	4.33	0.8357	0.5102
EM2	0.67	0.55	9.34			0.69	0.55	4.78		
EM3	0.60	0.64	7.72			0.56	0.68	3.70		
EM4	0.61	0.62	10.83			0.74	0.45	13.02		
EM5	0.87	0.24	13.84			0.89	0.21	12.36		
KL1	0.86	0.27	23.21	0.9455	0.7767	0.82	0.32	18.09	0.9298	0.7263
KL2	0.89	0.21	23.62			0.83	0.31	18.08		
KL3	0.93	0.13	24.45			0.90	0.20	19.30		
KL4	0.83	0.32	22.60			0.82	0.32	18.52		
KL5	0.90	0.19	24.03			0.89	0.22	15.29		
REP1	0.84	0.30	18.59	0.8841	0.6607	0.82	0.33	6.97	0.8829	0.6553
REP2	0.63	0.51	12.34			0.86	0.35	9.29		
REP3	0.92	0.16	14.74			0.89	0.21	8.33		
REP4	0.71	0.29	13.36			0.69	0.52	15.29		
HRG1	0.57	0.67	17.75	0.8441	0.5834	0.65	0.58	11.68	0.9338	0.7037
HRG2	0.67	0.55	6.04			0.88	0.23	6.25		
HRG3	0.64	0.59	6.41			0.88	0.22	6.19		
HRG4	0.51	0.74	5.78			0.87	0.24	5.88		
HRG5	0.89	0.20	6.82			0.90	0.19	6.37		
HRG6	0.81	0.34	6.68			0.83	0.32	6.38		
MINAT1	0.84	0.30	10.98	0.8075	0.5854	0.79	0.38	10.05	0.7510	0.5033
MINAT2	0.66	0.57	11.44			0.64	0.59	9.19		
MINAT3	0.79	0.38	12.70			0.69	0.52	9.71		

Remark: EM = BE (brand Equity), KL= SQ (service quality), REP = reputation, HRG = P (price), MINAT = I (intention).

Hypotheses test results done in UIN can be seen in Table 2 below. All of the hypotheses are supported. Brand equity and service quality affect the reputation positively and significantly. Likewise, reputation affects students’ intention to choose the university.

**Table 2.** Hypothesis test of Universitas Islam Negeri (UIN).

Hypothesis		SLF	T-statistic	Result
H1	BE ➔ REP	0.45	12.86	Positively significant
H2	SQ ➔ REP	0.85	24.63	Positively significant
H3	REP ➔ P	0.66	16.68	Positively significant
H4	REP ➔ I	0.76	18.81	Positively significant
H5	P ➔ I	0.63	16.10	Positively significant

Remark: BE (Brand Equity), SQ (Service Quality), REP (Reputation), P (Price), I (Intention).

Table 3 shows the Goodness of fit on UIN. All of the goodness of fit test results are in good fit. It means that all of the test results are over the standard.

**Table 3.** The goodness of fit of Universitas Islam Negeri (UIN).

Goodness-of-fit	Cut-off-Value	Result	Remark
RMR (Root mean square residual)	≤0.05 to ≤0.1	0.019	Good fit
RMSEA (Root mean square error of approximation)	≤0.08	0.021	Good fit
GFI (Goodness of fit)	≥0.90	0.99	Good fit
AGFI (Adjusted goodness of fit index)	≥0.90	0.98	Good fit
CFI (Comparative fit index)	≥0.90	0.98	Good fit
Normed Fit Index (NFI)	≥0.90	0.99	Good fit
Non-Normed Fit Index (NNFI)	≥0.90	0.99	Good fit
Incremental Fit Index (IFI)	≥0.90	0.99	Good fit
Relative Fit Index (RFI)	≥0.90	0.98	Good fit

Source: Statistic result.

The hypotheses test result of IIUM can be seen in Table 4 below. All the hypotheses are also supported positively and significantly, as well as UIN’s.

**Table 4.** Hypothesis test of International Islamic University Malaysia (IIUM).

Hypothesis		SLF	T- statistic	Result
H1	BE ➔ REP	0.59	13.58	Positive and significant
H2	SQ ➔ REP	0.79	19.58	Positive and significant
H3	REP ➔ P	0.44	9.47	Positive and significant
H4	REP ➔ I	0.80	9.10	Positive and significant
H5	P ➔ I	0.49	9.63	Positive and significant

Remark: BE (Brand Equity), SQ (Service Quality), REP (Reputation), P (Price), I (Intention).

The goodness of fit of IIUM is shown in Table 5 below. All of the goodness of fit test results are in good fit which is indicated by all of the test results are above the standard.

**Table 5.** Goodness of fit of IIUM.

Goodness-of-fit	Cut-off-value	Result	Remark
RMR (Root mean square residual)	≤0.05 to ≤0.1	0.016	Good fit
RMSEA (Root mean square error of approximation)	≤0.08	0.019	Good fit
GFI (Goodness of fit)	≥0.90	0.98	Good fit
AGFI (Adjusted Goodness of Fit Index)	≥0.90	0.97	Good fit
CFI (Comparative Fit Index)	≥0.90	0.97	Good fit
Normed Fit Index (NFI)	≥0.90	0.96	Good fit
Non-Normed Fit Index (NNFI)	≥0.90	0.98	Good fit
Incremental Fit Index (IFI)	≥0.90	0.97	Good fit
Relative Fit Index (RFI)	≥0.90	0.96	Good fit

Source: Statistic result.

## Discussion

### Effect of brand equity on the reputation of UIN and IIUM

Based on the statistical results, the brand equity of a university affects its reputation positively and significantly ( Arian M. and Tornike K., 2020). The item with the highest score that influenced the reputation of the two studied universities was the students’ recognition of their superiority. The item with the lowest score was the students’ immediate recognition of the logo of the universities. However, the effect of all item scores of brand equity on the universities’ reputations was positive and significant. Indonesian students recognized and knew well that UIN and IIUM are currently in high demand. The results show that the universities’ brand equity affects their reputation ( Ambler, 2003; Christodoulides and de Chernatony, 2010; Christodoulides *et al.*, 2015). The influence of brand equity on the reputation of IIUM was stronger than that of UIN.

### Effect of service quality on the reputation of UIN and IIUM

The results of the statistical tests showed that service quality affects the universities’ reputation positively and significantly ( Gronroos, C. *et al.*, 2019; Polkinghorne, M. *et al.*, 2017). Out of 19 question items posed to the respondents, the item with the highest score that influenced positively and significantly the reputation of UIN was the students’ belief that the university would meet their needs. It indicates that the highest score at UIN is in the reliability dimension, while for IIUM, the highest score was in the responsiveness dimension, *i.e.* its immediate response in serving the students. Usually, the reliability dimension is the most important dimension in a service firm such as a university ( Zeithaml, A. V. *et al.*, 2013). The item with the lowest score which influenced the reputation of UIN was its consistency in providing the timetable of its academic activities. While for IIUM, it was the neat appearance of its employees. These items’ scores were low because the students did not consider timetable information and appearance as the main factors needed to build a university’s reputation. However, answers to all the questionnaire items were positive and significant. Service quality had more effect on UIN than IIUM. It could be because the students believed that UIN can meet their needs. While at IIUM, although the responsiveness of the university was higher, the students did not consider service quality to be the most important thing.

### Effect of the universities’ reputation on students’ intention to choose the universities

The influence of service quality on reputation was stronger than that of brand equity. The universities’ reputation positively and significantly affected students’ intention to choose them ( Harahap *et al.*, 2018). The item with the highest score in this matter was the students who wanted to search for more positive information about the universities. The students intended to choose IIUM more than UIN. This could be a result of IIUM having a greater reputation than UIN, as seen in the results of the statistical tests. Therefore, it proves that the higher the reputation, the stronger the intention to choose a university. A good reputation is affected by the brand equity and service quality of the university.

## Conclusions

This is the first research that compares Indonesian students’ intention to choose a university with the same concept in Indonesia and Malaysia. The study results show that the brand equity and service quality of a university affect its reputation. In turn, reputation affects students’ intention to choose a university. The higher the reputation of a university, the stronger will the students’ intention to choose it be. This is the first research conducted in this area to the best of the researchers’ knowledge. Hence, it might help universities in Indonesia to enhance their brand equity and service quality to gain a higher reputation, since reputation is an important factor that influences students’ intention to choose a university. This will cause more students to enroll in the universities. The findings also provide a reference for UIN and IIUM, and especially PNJ in making strategies to increase their reputations and students’ intention to choose them.

## Data Availability

Figshare: Raw Data result of survey, https://doi.org/10.6084/m9.figshare.19826404 ( Rozza, 2022a) This project contains the following underlying data:
- Raw data for F1000 update.csv Figshare: Questionnaire Research, https://doi.org/10.6084/m9.figshare.20506776 ( Rozza, 2022b) Data are available under the terms of the Creative Commons Zero “No rights reserved” data waiver (CC0 1.0 Public domain dedication).
