# Supplementary material for: Impact of brand equity and service quality on the reputation of universities and students’ intention to choose them: The case of IIUM and UIN
Source: F1000Res. 2023 Oct 5;11:1412. Originally published 2022 Nov 30. [Version 3] doi: 10.12688/f1000research.122386.3 (PMC10521097; doi:10.12688/f1000research.122386.3)
Supplement: Peer review history

# Reviewer report: Reviewer response for version 2

**DOI:** 10.5256/f1000research.154830.r201234

**Published:** 2023-09-15

**Authors:** Mochammad Riyadh Rizky Adam

**Affiliations:** 1 Management, Faculty of Business and Accountancy, Lincoln University College, Petaling Jaya, Selangor, Malaysia; 2 Business and Management, Faculty of Business & Communications, Swiss German University, Alam Sutera, Tangerang, Indonesia

Esteemed authors and researchers,

 I would want to express my gratitude for extending another invitation to me to evaluate the revised manuscript. I can observe a significant amount of progress that facilitates the readers' comprehension.

 There are two suggestions that I would like to propose.

 (1) Firstly, it is recommended to exclude the sentence "...which is moderated by study expense (price)" from the abstract, as this aspect will be examined and further elaborated in the forthcoming study conducted next year.

 (2) Since the authors/researchers have the raw data, I suggest to re-run the SEM analysis again without 'Study Expense (Price)' as the moderating variable. Keep it in the next year's study to keep the surprise or new thing of the study beside it is not common either. Do not show/display in this current study (including the hypothesis development part).

 Apart from the above, I have no further comments to give. 

 Thank you.

Is the work clearly and accurately presented and does it cite the current literature?

Partly

If applicable, is the statistical analysis and its interpretation appropriate?

Yes

Are all the source data underlying the results available to ensure full reproducibility?

Partly

Is the study design appropriate and is the work technically sound?

Partly

Are the conclusions drawn adequately supported by the results?

Yes

Are sufficient details of methods and analysis provided to allow replication by others?

Partly

Reviewer Expertise:

Marketing, branding, social marketing, green marketing, sustainable marketing, cause-related marketing, digital marketing, social media marketing.

I confirm that I have read this submission and believe that I have an appropriate level of expertise to confirm that it is of an acceptable scientific standard.

# Reviewer report: Reviewer response for version 2

**DOI:** 10.5256/f1000research.154830.r201235

**Published:** 2023-08-30

**Authors:** Yuen-Onn Choong

**Affiliations:** 1 Faculty of Business and Finance, University of Tunku Abdul Rahman, Kuala Lumpur, Malaysia

Thank you for giving me an opportunity to review the revised version of the manuscript. I am happy to see the paper which with lot of improvement by considering the comments given in the previous review. Thus, I have no further comments.

Is the work clearly and accurately presented and does it cite the current literature?

Yes

If applicable, is the statistical analysis and its interpretation appropriate?

Yes

Are all the source data underlying the results available to ensure full reproducibility?

Yes

Is the study design appropriate and is the work technically sound?

Yes

Are the conclusions drawn adequately supported by the results?

Yes

Are sufficient details of methods and analysis provided to allow replication by others?

No

Reviewer Expertise:

Educational management, leadership, behavioural studies

I confirm that I have read this submission and believe that I have an appropriate level of expertise to confirm that it is of an acceptable scientific standard.

# Response

**Published:** 2023-08-31

**Authors:** Dr. Sylvia Rozza SE MM Rizard

**Affiliations:** POLITEKNIK NEGERI JAKARTA, Indonesia

Thank you very much Mr. Juen-Onn Choong for the approval of the new version of my article. I hope this will accelerate the indexing process.

 Regards,

 Sylvie

# Reviewer report: Reviewer response for version 1

**DOI:** 10.5256/f1000research.134367.r185234

**Published:** 2023-07-25

**Authors:** Mochammad Riyadh Rizky Adam

**Affiliations:** 1 Management, Faculty of Business and Accountancy, Lincoln University College, Petaling Jaya, Selangor, Malaysia; 2 Business and Management, Faculty of Business & Communications, Swiss German University, Alam Sutera, Tangerang, Indonesia

Thank you for inviting me to review the research paper, titled "Impact of brand equity and service quality on the reputation of universities and students’ intention to choose them: The case of IIUM and UIN". Here are some concerns that need more work:

**Several issues with the abstract section:**

- The novelties that the paper brought are not visible to the readers.
- After the author said “Numerous studies have been carried out on the impacts of brand equity and service quality of higher education institutions (HEIs) on their reputation and students’ satisfaction…..”, it would be best and clear if the authors show the gap between existing literature and the things/situation that happening in the higher education industries.
- Many misunderstandings. The abstract makes the claim that the study expense (price) acts as a moderator, yet this is at odds with the research framework presented on page 6. In the SEM measurement model illustrations, however (pages 7-9), the price variable clearly plays the role of a mediator (rather than a moderator). In addition to these, it is stated that the limitation of the study is the effect of study expense on the intention to study has not been conducted. However, we can see that the study examined the relationship between the study expense and the intention to study for both universities.
- Key information in the research methodology were not displayed well. Several other missing important information: research type, sampling method, data method of obtaining data.

**Several issues with the introduction section:**

 The structure of the writing should be improved more to give good flow of the understanding so that the readers will understand what happens in Indonesia and Malaysia, why such investigation (comparing Indonesia and Malaysia) is crucial to be done (Can't the authors find the answers of the research questions from the existing empirical research papers?). It is also important to provide solid reason why brand equity, service quality, reputation, and study expense (price) are important factors that happens in the higher education especially in the discussion of intention to study in the Islamic-based and Non-Islamic-based universities. Several details comments as follow: 

- It's important to differentiate between universities founded on Islamic principles and those that weren't from the outset, thus distinct terms should be used to describe each. The authors may differentiate between Islamic universities and non-Islamic universities to avoid confusion. As the example, the authors wrote with this statements: "The UIN format allows universities to open faculties or departments in other fields of study outside of Islamic studies. The impact of these changes is the increase in the number of universities that offer majors in faculties of non-Islamic studies that can be chosen by prospective students."
  I think it would be clear if stated with this way "The UIN format allows Islamic-based universities to open faculties or departments in other fields of study outside of Islamic studies. The impact of these changes is the increase in the number of Islamic-based universities that offer majors in faculties of non-Islamic studies that can be chosen by prospective students." 
- Precise justification needed to support the thought that "The number of students who are interested in studying at UIN continues to increase, while the number of students who enrolled at the University of Indonesia (UI), the biggest competitor of UIN, has decreased". It would be difficult for the readers who are interested to explore further the justification (by clicking the provided link) as the link is kind of a do-it-yourself-find-yourself page. 
- If the authors claim no similar research framework already exists in the literature, the authors should provide stronger and clear explanations and evidences for the claim. From the paper, it is clear to the readers that research into the relationship between brand equity-service quality and reputation has been conducted.
- The above comment leads to this concern: the research gap should be explained well. So that the readers will understand the existing literature lack of. The readers will also identify the novelty(ies) easily.
- "The researchers have not been able to find research in Indonesia that examines the impact of a university’s reputation based on its brand equity and service quality on students’ intention to choose it".- How about from other countries research papers? From other context/industry?

**Several issues with the literature section:**

- Is there a theory that underpins the entire research model? If so, the readers would want to know about it in the literature section.
- There is no explanation about the research framework. Please explain and elaborate it clearly in the literature section
- The proposed hypotheses should have been explained with strong justifications (from existing empirical research papers and/or qualitative research if any/conducted). Please provide the explanation and justification how the proposed hypotheses were developed.
- If the measurement model displays study expense (price) as the mediating variable, does study expense (price) have a positive or negative effect on the intention to study? The answer will affect how the raw data of study expense (price) will be processed (converted to the opposite scale code or not).

**Several issues with the methodology section:**

- The methodology part should be re-written in order to give good understanding how the authors conducted the study.
- Minimum information to be provided: research type, targeted population, data collection method, sampling method, sampling size (the round up does not make sense), research instruments/questionnaire explanation (screening question, questionnaire structure, measurement items and scales), back translation technique used or not, data analysis techniques including with the software used (did the authors do any test before the SEM such as multivariate assumption test?).
- Since it will be biased if we ask about the intention to choose/to buy/to purchase to someone who has chosen/enrolled/purchased already, the authors should explain why they distributed the questionnaire to respondents who have become students already (for both UIN and IIUM). There should be an explanation (in the literature section) with strong justifications that the intention to choose/to buy/to purchase is the same as the intention to re-choose/to re-buy/to re-purchase.
- If the intention to choose/to buy/to purchase is the same as the intention to re-choose/to re-buy/to re-purchase, the authors need to put the context how the respondents who have become the students asked about the intention to choose. Show the measurement items of the intention to choose would be great for the readers.
- Were the questionnaire questions written in a retrospective question/statement manner, given that the respondents asked were the ones who have been students already? If this approach was taken, the authors must give the reason why this method was taken, given that the dependent variable was intention, not purchase decision.
- It's good also if the methodology section can be explained in a table, displaying the whole process/stages done or conducted by the authors.
- It would be helpful if the authors detailed the procedures used on the pretest sample (taken before the final/big sample was collected, if any).
- It would be helpful if the authors mentioned the software used in running the SEM analysis.

**Several issues with the results section:**

- It would have been helpful to see profiles of the respondents in terms of the demographics.
- The results part can be re-structured in order to give good flow and capture the whole process done. This does not mean the authors have to explain in detail, but the authors could tell what process that has been done with the key results only (these can be displayed in a/several tables) to inform the readers that the data have actually through multiple examinations before jumping to the SEM or hypotheses testing results. 
- Since there are H3 (Reputation to Price) and H5 (Price to Intention), the role Price needs to be clear whether as mediating variable or moderating variable between a relationship (is it between reputation to intention? This answer will affect the results and discussion. 

**Several issues with the conclusion section:**

- It would be great for the readers to know the implications of the study. Is there any implications for the industry, for the companies (what recommendations the authors suggest to do according to the results) or for the theory development.
- Is there any recommendations or path that can be taken for further research?

**Several issues with the references:**

- If the literature review was revised, it would include more sources.
- Use the recent references.

Is the work clearly and accurately presented and does it cite the current literature?

Partly

If applicable, is the statistical analysis and its interpretation appropriate?

Yes

Are all the source data underlying the results available to ensure full reproducibility?

Partly

Is the study design appropriate and is the work technically sound?

Partly

Are the conclusions drawn adequately supported by the results?

Yes

Are sufficient details of methods and analysis provided to allow replication by others?

Partly

Reviewer Expertise:

marketing, branding, social marketing, green marketing, sustainable marketing, cause-related marketing, digital marketing, social media marketing.

I confirm that I have read this submission and believe that I have an appropriate level of expertise to confirm that it is of an acceptable scientific standard, however I have significant reservations, as outlined above.

# Reviewer report: Reviewer response for version 1

**DOI:** 10.5256/f1000research.134367.r176120

**Published:** 2023-06-09

**Authors:** Yuen-Onn Choong

**Affiliations:** 1 Faculty of Business and Finance, University of Tunku Abdul Rahman, Kuala Lumpur, Malaysia

Thank you for allowing me to review the paper entitled " Impact of brand equity and service quality on the reputation of universities and students’ intention to choose them: The case of IIUM and UIN" Here are some suggestions to improve the paper:

 1. Originality: Does the paper contain new and significant information adequate to justify publication?

1. The current version of the abstract is not well written. There is some important detail of the study is not mentioned in the abstract. First of all, it should start from the main aim of the study and tell a little bit about the novelty of this study. This followed by the research method such as sample size, method/technique used to test the hypotheses and model. Then end with the key findings and implications of the study. What’s new to this study?
2. I have noticed that the introduction lacks a well-structured and cohesive argument regarding the inclusion of brand equity and service quality in this study and its relationship to reputation and satisfaction. The authors have not clearly articulated why this concept is being investigated and what significance it holds. It is important for them to provide a clear picture and rationale behind the study of reputation and satisfaction. Furthermore, it would be beneficial for the authors to address any potential shortcomings or limitations associated with the concept of the study in order to strengthen their argument.
3. Authors have highlighted several past studies. Authors also claimed that there are many studies examined all these variables. Thus, authors fail to explain how and what these studies lack of. By doing so, they can establish a foundation and context for their own research. Subsequently, the authors can explain the gaps or limitations that exist in the previous studies, providing insights into how their own study differs and addresses those gaps. This approach will help the authors identify the novelty and unique contribution of their research. Is the comparison between two universities is one of the issues? Is this worth to know the differences?
4. In addition, it is important for the authors to emphasize the urgency of their study and clearly articulate how it relates to the key concepts. They should outline the significance and potential implications of their research, highlighting why it is important to explore all these key concepts within the context of the study. By clearly expressing the relevance and timeliness of their work, the authors can engage readers and establish a compelling rationale for conducting the research.

2. Relationship to Literature: Does the paper demonstrate an adequate understanding of the relevant literature in the field and cite an appropriate range of literature sources? Is any significant work ignored?

1. Should have a standalone section for underpinning theory to explain how the path relationships work.
2. Hypotheses should be proposed in the literature review. Should also talk about the interrelationship between the variables.
3. The propose conceptual framework should be presented in the literature review section.

3. Methodology: Is the paper's argument built on an appropriate base of theory, concepts, or other ideas? Has the research or equivalent intellectual work on which the paper is based been well designed? Are the methods employed appropriate?

1. The methodology section needs substantial improvement. To enhance clarity and understanding, it is recommended to divide the section into two subsections: (1) research procedure and sample, and (2) research instruments. This division will provide a clear structure and allow readers to easily navigate the methodology section.
2. To improve the paper, it is important to clearly define the context of the study and provide strong justifications for the chosen target respondents. Provide a concise and clear explanation of the context in which the study is conducted.
3. Any translation conducted for the adopted instrument? What translation method used?
4. Discuss the generalizability and representativeness of the sample in relation to the target population. Clearly explain how the chosen sample is intended to be representative and reflect the larger population. Highlight any strategies employed to ensure a diverse and inclusive sample.
5. It is important to clearly explain the sampling technique used in the study, along with the rationale for its selection. Additionally, the authors should describe how they used this sampling technique to select respondents for the survey, ensuring generalizability and representativeness towards the targeted population.
6. What do you mean round up to 228? I though round up mean you should round up to 230 or 250.
7. What software used in this study? Provide adequate justification.
8. The procedure of data collection is too limited. More information should be provided on how the authors collected the data, approached the respondents, and identified them to participate in the survey. The explanation should be logical and reasonable, without exaggeration.
9. Including control variables in the study is indeed a good practice as they can help account for potential confounding factors that may influence the results. To improve the paper, it is important to include control variables and provide reasonable justifications for their inclusion.

4. Results: Are results presented clearly and analysed appropriately? Do the conclusions adequately tie together the other elements of the paper?

1. Demographic profile or characteristic of sample is very limited. Should report more.
2. The abundance of study results presented has made it challenging to comprehend and digest the findings. The extensive presentation of these results has resulted in a loss of interest and motivation to continue reading the paper. Authors should present the result in a more systematic way and organised. Simplify your result instead of giving so many figures and tables to made reader confuse and difficult to understand.

5. Practicality and/or Research implications: Does the paper identify clearly any implications for practice and/or further research? Are these implications consistent with the findings and conclusions of the

1. The discussion and conclusion are required substantial improvement.
2. The current version of the discussion is too shallow. The authors need to ensure that the key findings are discussed. The discussion section is where you delve into the meaning, importance, and relevance of your results. It should focus on explaining and evaluating what you found, showing how it relates to your literature review and research questions, and making an argument in support of your overall conclusion.
3. There should be a standalone section for theoretical implications. How can you imply from the findings? This section should discuss the implications of the study's findings and how they contribute to the existing theoretical knowledge. Summarize the key findings and their relevance to the existing theoretical frameworks or models. Analyze how the findings align with or challenge current theoretical perspectives and concepts related to all the key concepts of this study. Discuss any theoretical insights or advancements that the study provides and highlight how the findings contribute to a deeper understanding of the research area.
4. There should be a standalone section for practical implications. I would suggest the author provide implications based on current practices and policies.
5. The current form of limitation and future research recommendation section is not well written. In this section, the authors should conscientiously identify the potential weaknesses or limitations of the study and provide insightful suggestions for future research directions. By incorporating this standalone section, the paper will not only address its own limitations but also serve as a valuable resource for researchers seeking to build upon the current study and expand the knowledge base in the field.
6. Should have a standalone section for limitation and future research recommendation section. In this section, the authors should conscientiously identify the potential weaknesses or limitations of the study and provide insightful suggestions for future research directions. By incorporating this standalone section, the paper will not only address its own limitations but also serve as a valuable resource for researchers seeking to build upon the current study and expand the knowledge base in the field.

6. Quality of Communication: Does the paper clearly express its case, measured against the technical language of the field and the expected knowledge of the journal's readership? Has attention been paid to the clarity of expression and readability, such as sentence structure, jargon use, acronyms, etc.

1. Add more recent citations.
2. Required professional proofreading and editing services.

Is the work clearly and accurately presented and does it cite the current literature?

Yes

If applicable, is the statistical analysis and its interpretation appropriate?

Yes

Are all the source data underlying the results available to ensure full reproducibility?

Yes

Is the study design appropriate and is the work technically sound?

Yes

Are the conclusions drawn adequately supported by the results?

Yes

Are sufficient details of methods and analysis provided to allow replication by others?

No

Reviewer Expertise:

Educational management, leadership, behavioural studies

I confirm that I have read this submission and believe that I have an appropriate level of expertise to state that I do not consider it to be of an acceptable scientific standard, for reasons outlined above.
